# Persistence and remission of depressive symptoms and psycho-social correlates in Chinese early adolescents

**DOI:** 10.1186/s12888-020-02808-5

**Published:** 2020-08-12

**Authors:** Xinli Chi, Benjamin Becker, Qian Yu, Md Mahhub Hossain, Jingyaun Lin, Albert Yeung, Radhika Seiler-Ramadas, Igor Grabovac, He Bu, Fei Xie, Liye Zou

**Affiliations:** 1 Center for Lifestyle and Mental Health, School of Psychology, Shenzhen University, Shenzhen, 518060 China; 2 University of Electronic Science and Technology of China, Chengdu, 610054 China; 3 Exercise & Mental Health Laboratory, School of Psychology, Shenzhen University, Shenzhen, 518060 China; 4 Texas A&M University, College Station, TX 77843 USA; 5 Massachusetts General Hospital, Harvard Medical School, Boston, MA 02114 USA; 6 Medical University of Vienna, Kinderspitalgasse 15/1, 1090 Wien, Austria; 7 Department of Social and Behavioural Sciences, City University of Hong Kong, Hong Kong, China; 8 School of Nursing, Army Medical University, Chongqing, 400038 China

**Keywords:** Persistent depressive symptoms, Positive youth development, Family functioning, Chinese adolescents

## Abstract

**Background:**

This study aimed to investigate a one-year course of persistent/remitted depressive symptoms and associated demographic and psychosocial factors that predict persistent/remitted depressive symptoms in Chinese high school students.

**Methods:**

One thousand five hundred forty-four Grade 7 students provided data for the first wave. Of the initially recruited students, 483 who were classified as depressed (CESD score ≥ 16) at baseline were then tracked and invited to fill in the questionnaire for a second time (Grade 8) after 1 year. Finally, 435 of them were successfully matched.

**Results:**

Two hundred two (46.4%) of the subset categorized as depressed in the first survey (*N* = 435) remained with depressive symptoms, while 233 (53.6%) recovered from depression 1 year later. Having siblings, a lower level of positive youth development, non-intact family status, and poor family functioning at baseline significantly predicted a higher likelihood of persistent depression, while those with fathers having higher educational qualifications (bachelor’s degree or higher) at baseline showed a significantly higher probability of remitting from depression.

**Conclusions:**

The findings indicated that the prevalence of persistent depressive symptoms was generally high, and promoting aspects of positive youth development and family functioning for adolescents could be promising in preventing or reducing these symptoms.

## Background

Depression is one of the leading causes of adolescent illness and disability, which is associated with serious detrimental consequences such as social and educational dysfunction, substance abuse, aggression, bingeing, and high risks of suicide [1]. As a result, depression not only impairs the normal psychosocial development of the adolescents, but also places a substantial financial burden (juvenile justice and education programs, for example) on the family of depressed adolescents and the society [2]. Earlier observational studies found high prevalence rates of depression among adolescents alongside several potential risks and/or protective factors [3–5]. However, due to the cross-sectional design of previous studies, in which data are gathered only at single time points, the persistence of depressive symptoms and the effects of risk and/or protective factors on the course of depressive symptomatology in adolescence remain largely unknown. Notably, factors that contribute to the onset of depressive symptomatology in adolescence may differ from those that lead to the persistence of depression. Therefore, exploring the temporal trajectory of adolescent depression may lead to a more robust and accurate determination of risk factors than a single-time assessment of depressive symptoms [6, 7]. Given the high prevalence rates and detrimental impacts of adolescent depression, a better understanding of persistent depressive symptoms during adolescence and associated risks and/ or protective factors is urgently needed. Moreover, the results can be used by key stakeholders including the policy-makers, health professionals, and parents, to identify subjects vulnerable to persistent depression and to develop more effective early interventional approaches to prevent the same.

The prevalence rates of persistent depressive symptoms in adolescents are found to vary greatly across countries, regions, and populations. For example, one study conducted in the USA included 1176 adolescents who reported depressive symptoms at baseline and indicated that 38.5% of them exhibited persistent depressive symptoms within a 12-month follow-up period [8]. In addition, many longitudinal studies with different follow-up periods (6-months, three-years, and 10-years) conducted in Switzerland, the UK, and Germany reported early and late adolescents with either depression alone [9, 10] or depression with coronary heart disease [11]. In these studies, the baseline and post-assessment of persistent depressive symptoms were reported with prevalence rates ranging from 5 to 12.2%. Likewise, Chinese scholars recently conducted one longitudinal study where they focused on middle school adolescents. The study conducted in Guangzhou included 5365 students, with 735 that were reported with depression at baseline and 57.14% of initially self-reported depressed participants having remained with symptoms after 9 months [12]. Admittedly, the results of the aforementioned studies provide valuable information as the majority of studies that have investigated the prevalence of persistent depression in adolescents were conducted in Western countries (e.g., USA and Europe). However, depression among Chinese adolescents from metropolitan areas like Shenzhen, and those who study in highly competitive academic environments remains poorly investigated. As such, we conducted a two-wave longitudinal study with data collected at baseline and an interval of 1 year to investigate the prevalence of persistence/remission of depressive symptoms in Chinese adolescents.

Moreover, to prevent adolescent depression at an early stage, it is essential to identify potential factors that may have contributed to depression in this age group. Based on developmental psychopathology perspective [13, 14], the development of depression in youth was jointly influenced by genetic, biological, psychological, and social factors. Of them, demographic, psychological, and familial factors were very likely to have influences on the onset and persistence of adolescent depression [15, 16]. Using developmental psychopathology framework, we focused on whether personal factors including demographics (i.e. age, gender, sibling [presence or absence], migrant status), psychological factor (i.e., positive youth development), and familial factors (i.e., parents’ education level, family structure, and family function) are related to the persistence of depression among Chinese adolescents.

Empirically, it was reported that adolescents were more susceptible to depression with increasing age [17], if they were biologically female, migrant students [18], had sibling(s), and/or a non-intact family (e.g., divorced families and single-parent families) [19, 20]. On the other hand, higher positive youth development (PYD) (e.g., self-efficacy, resilience, and positive identity) and greater parental educational attainment [21, 22] have been well-documented to serve as a protective factor against negative developmental outcomes like depression [23]. Furthermore, there has been accumulating evidence supporting the idea that family functioning plays a major role in the development of depression [24]. Family functioning generally refers to the global family environment in terms of socialization and structure, including levels of harmony, relationship and interactions within the family, levels of conflict and cohesion, organization, and the quality of communication. Lower levels of family functioning were found to associate with a greater likelihood of developing adolescent depression [25–27]. Based on this earlier evidence, we hypothesized that older adolescent age, females, having sibling(s), migrant background, non-intact family, lower positive youth development, poor family functioning, as well as lower parental educational attainment, are factors that render adolescents more susceptible to persistent depressive symptoms.

Against this background, the present study had two aims. The first aim of the study was to examine the prevalence of persistent depressive symptoms over 1 year among Chinese adolescents. The second aim was to explore personal predictive factors (i.e., gender, age, sibling (presence or absence), migrant status, and positive youth development) and family predictive factors (i.e., parents’ education level, family structure, and family function) of persistent depressive symptoms among Chinese adolescents.

## Method

### Participants

This study was conducted in Shenzhen city, which is a major metropolitan area in South China. Chinese students were recruited from junior secondary schools, and two surveys were completed within 1 year. During the study period in 2018, there were 1,476,000 students within more than 150 public junior high schools located in Shenzhen (Shenzhen Education Bureau, 2019). Of these public schools, six were randomly selected between October and November 2016, with 1544 Grade 7 students providing data for the first wave. Of the initially recruited students, 483 were classified as depressed (CESD score > = 16) at baseline and were then tracked and invited to fill in the questionnaire for a second time (Grade 8) between October and November 2017. Students who missed the school day in which the second survey was conducted or failed to put their identification numbers on the questionnaires were excluded. Finally, only 435 initially self-reported depressed participants were matched successfully with each participant’s school number, implying an acceptable attrition rate of 9.9%. The mean age of the participants in this study at baseline was 12.46 (SD = .65).

### Procedure

Participants were invited to complete multiple questionnaires on PYD, family functioning, depression, and social-demographic characteristics. Data collection was administered by two trained graduate students who gave standardized instructions within the classroom settings. One graduate student introduced the purpose of this study, while the other helped maintain classroom order. Students were required to sit separately, to not speak, and to not engage in discussion. The duration of the survey was approximately 20 min. There was the consideration of the high levels of stigma against having a psychiatric disorder among Chinese and high degree of privacy [28]. All students and parents were informed at the time of consent. Procedures are detailed as follows. Firstly, the informed consent form was sent to principals of all surveyed schools for their approval and then forwarded to teachers who were in charge of their respective classes. All responsible teachers had distributed the important document to students and asked for voluntary signature(s) of their parents or legal guardian(s). The purpose of this study was clearly explained in the consent form. We only handed out questionnaires those children of parents(s) or legal guardian(s) who signed the consent form. The data collected would not be looked at individually and would be analyzed in an aggregated manner at the start of each session. Subsequently the data was published in a research paper with personal information being kept strictly confidential. The two trained graduate students were present throughout the administration process to answer participants’ questions. This study was approved by the Human Research Ethics Committee of Shenzhen University and all principals of the surveyed schools.

### Measurements

#### Sociodemographic correlates

Participants were invited to report on their age, gender (1 = male and 2 = female), and whether they were from a one-child family (1 = one child, 2 = non-one child). They also reported their parental education levels (1 = less than or middle school, 2 = high school and college, 3 = university, and 4 = more than university) and whether they grew up in an intact family (coded as 1) or a non-intact family (coded as 2). The demographic data of the study participants are presented in Table 1.

**Table 1:** Characteristics of depressed participants at baseline (*N* = 435)

Predictors	n/M	%/SD
***Individual factors***		
Age	12.46	.65
Gender		
Male	210	48.3
Female	220	50.6
Missing	5	1.1
Siblings		
One Child	152	34.9
Non-one child	283	65.1
Missing	0	0
Migrant status		
Migrant students	100	23.0
Local students	332	76.3
Missing	3	.7
Positive Youth Development	4.36	.70
***Familial factors***		
Family intactness		
Intactness	399	91.7
Non-intactness	32	7.4
Missing	4	.9
Father’s Education Level		
Less than or middle school	156	35.9
High school or college	142	32.6
University	70	16.1
More than university	37	8.5
Missing data	30	6.9
Mother’s Education Level		
Less than or middle school	192	44.1
High school or college	124	28.5
University	63	14.5
More than university	29	6.7
Missing data	27	6.2
Family Function	3.72	.80

#### Dependent variable

##### Depression

The 20-item Center for Epidemiologic Studies Depression Scale (CES-D) was utilized to assess levels of depression, which is validated for Chinese population [29, 30]. Participants were asked to rate how often they experienced symptoms related to depression in the past 7 days using a four-point Likert scale (0 = rarely or none of the time, 3 = most or all of the time). The scores for each item were added up, which resulted in the 20-item sum score of a maximum of 60 (greater scores indicated a higher level of depressive symptoms). Notably, participants who scored less than 16 were healthy, whereas those who scored 16 or higher (CES-D ≥ 16) were considered as depressed [31, 32]. Prior studies have shown that the CES-D has good psychometric properties [33, 34]. The Cronbach’s alphas for the present study were > .85 at each of the two assessments.

#### Independent variables

##### PYD

The Chinese Positive Youth Development Scale (CPYDS) was used to measure 15 positive youth development constructs such as resilience, self-efficacy, and emotional competence [35, 36]. Example items on this scale include, “*I am a filial person*” and “*I know my strengths and weaknesses*”. Participants were asked to select one of four options which best fit their own circumstance based on a four-point Likert scale (0 = *strongly disagree*, 3 = *strongly agree*). In the present study, the average of the total score for CPYDS was adopted as an indicator of personal positive youth development, with higher scores representing higher psychological competencies. The CPYDS has demonstrated reliability and validity in the sample of Chinese adolescents in previous studies [35, 36]. The Cronbach’s alphas were > .95 at each of the two assessments.

##### Family functioning

The Chinese Family Assessment Instrument (CFAI) that consists of 9 items was adapted to assess the general family function [37]. Example items on this scale include, “*We don’t get along well together*” and “*We confide in each other*”. Participants were asked to select one of five options, which best fit their own condition on a 5-point Likert scale (1 = *very similar*, 5 = *very dissimilar*). The mean score was used to reflect family function, with higher mean scores representing better family functioning. The shortened 9-item CFAI has shown acceptable internal consistency in previous studies [38, 39]. The Cronbach’s alphas were > .85 at each of the waves of assessment in the present study.

### Statistical analyses

Firstly, frequencies and percentages were computed to examine the one-year prevalence of persistent and remitted depressive symptoms among Chinese adolescents. The percentages of adolescents whose CES-D scores indicated depression (≥16 points) and those who recovered from depressive symptoms (< 16 points) were computed. Secondly, Chi-square tests and independent sample t-tests were conducted to compare whether there were differences in the characteristics of participants between persistence of depression and remission of depression within 12 months. Thirdly, two multilevel logistic regression analyses (level 1: individual; level 2: familial) were conducted to examine the over-time predictive effects of independent variables on persistence/remission of depression across 12 months **(**Table 2**)**. Depressive symptoms (persistent depressive symptoms were coded as 1; remitted depressive symptoms were coded as 0) at wave 2 were considered as the dependent variable. Independent variables at baseline, including age, gender, sibling presence, migrant status, level of positive youth development, were put in the first block (level 1), while parental educational level, family structure (family intactness), and family functioning were put in the second block (level 2). Poisson distributions were normally approximated to calculate the 95% confidence intervals (CI). Two-tailed test with a *p* value of less than .05 was used to determine the level of significance, using SPSS Version 23.0.

**Table 2:** Multilevel logistic regression modelling results for persistence of depression across one year

Predictors	Model 1		Model 2	
	***B***	OR and 95% C.I.for OR	***B***	OR and 95% C.I.for OR
***Individual factors***				
Age	−.09	.91 (.66, 1.26)	−.14	.87 (.62, 1.22)
Gender		1		
Male		1.01 (.66, 1.53)		1
Female	.10		.07	1.08 (.70, 1.67)
Siblings				
One Child		1		1
Non-one child	.79	2.19 (1.40, 3.44) **	.74	2.10 (1.27, 3.48) *
Migrant status				
Migrant students		1		1
Local students	−.13	.88 (.53, 1.44)	.03	1.03 (.61, 1.73)
Positive Youth Development	−.39	.67 (.50, .92) *	−.29	.74 (.53, 1.05)
***Familial factors***				
Family intactness				
Intactness				1
Non-intactness			1.05	2.87 (1.16, 7.12) *
Father’s Education Level				
Less than or middle school				1
High school or college			−.26	.77 (.45, 1.32)
University			−.35	.71 (.31, 1.61)
More than university			−1.46	.23 (.07, .76) *
Mother’s Education Level				
Less than or middle school				1
High school or college			.08	1.08 (.63, 1.86)
University			.31	1.36 (.56, 3.27)
More than university			.86	2.37 (.68, 8.25)
Family Function			−.30	.74 (.54, 1.01) *
−2 × log likelihood	513.33		494.88	
Cox & Snell R^2^	.04		.09	
Nagelkerke R^2^	.06		.12	

*p < .05, **p < .01, ***p < .001

## Results

### Characteristics of participants at baseline

As seen in Table 1, 50.6% were female, 65.1% had sibling(s), and 76.3% were local children. Around one-third of the participants reported that parents’ educational levels were high school or college education (father: 32.6%; mother: 28.5%). The majority of students (91.7%) were from intact families. Participants generally reported a high level of positive youth development (4.36 ± .70) and good family functioning (3.72 ± .80).

### Prevalence of persistent depression among the participants over one year

**Fig. 1:**
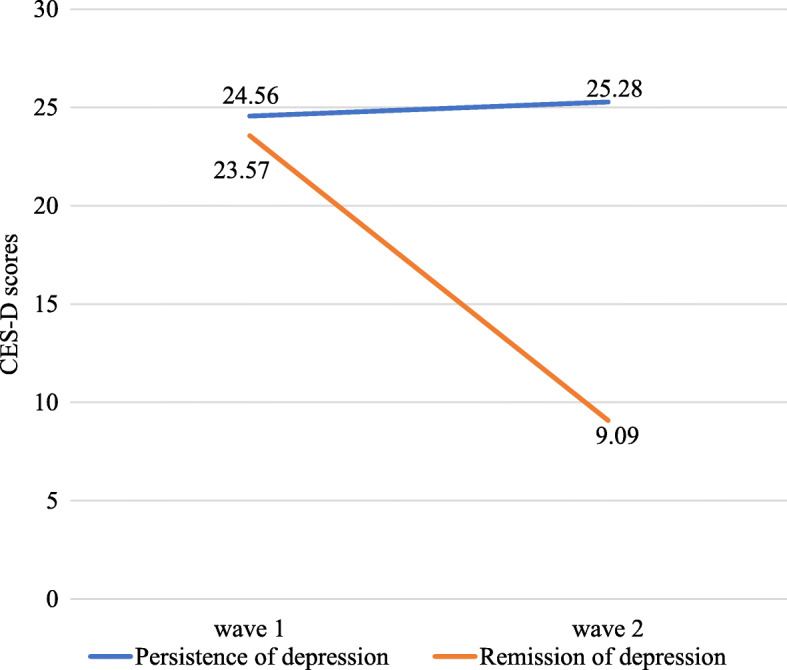
Continuous scores on CES-D over two waves of data

As shown in Table 3, 435 successfully matched adolescents at baseline reported depression with the CES-D sum score of ≥16). After 12 months, 202 (46.4%) of the initially self-reported depressed participants remained with depressive symptoms, while 233 (53.6%) of them recovered from depression. Regarding continuous scores on CES-D over two wave of data, the mean score of CES-D at persistent depression group was 24.56 (SD = 7.40, range from 16 to 50 points) at baseline and 25.28 (SD = 7.43, ranging from 16 to 55 points) (Fig. 1). The mean score of CES-D at remitted depression group was 23.57 (SD = 7.21, ranging from 16 to 51 points) at baseline and 9.09 (SD = 4.06, ranging from 0 to 15 points) (Fig. 1).

**Table 3:** Prevalence of persistent and remitted depressive symptoms across 12 months

	Prevalence of depressed participants at baseline	12- month persistence	12- month remission
	n	n/%	n/%
All depression (≥16 points)	435	202/46.4	233/53.6

### Predictors of persistent depression among the participants over one year

In the univariate analysis, five variables (sibling presence, positive youth development, family structure/intactness, educational attainment, and family functioning) were observed to significantly correlate with persistent depression (all *p* values < .05). However, age, gender, and migrant status of the participants were not significantly related to their persistent/remitted depressive symptoms (all *p* values > .05). Furthermore, Multilevel logistic regression showed that on the individual level, presence of siblings (OR = 2.19; 95% CI: 1.40, 3.44) and lower positive youth development at baseline (OR = −.67; 95% CI: 0.50, 0.92) were significantly associated with a higher risk of developing persistent depressive symptoms (Table 2). Age, gender, and migrant status at baseline were not significantly associated with the persistence of depression. For the familial level, students who lived in a non-intact family (OR = 2.87; 95% CI: 1.16, 7.12) and with worse family functioning had a significantly greater likelihood of developing persistent depression (OR = 0.74; 95% CI: 0.54, 1.10). Those whose father had a high educational level (bachelor’s degree or above) experienced a protective effect, with a higher probability of remitting from depression (OR = -0.23; 95% CI: 0.07, 0.76) (Table 4).

**Table 4:** Individual and familial characteristics of participants between remission of depression and persistence of depression across12 months (*N* = 435)

Predictors	Across 12 months		***t/x***^***2***^
	Remission of depression (***N*** = 233)	Persistence of depression (***N*** = 202)	
	n/% (M/SD)	n/% (M/SD)	
***Individual factors***			
Age	12.47/.64	12.47/.67	.08
Gender			.83
Male	118/50.6	92/45.5	
Female	114/48.9	106/52.5	
Missing	1/.4	4/2.0	
Siblings			11.18 **
One Child	98/42.1	54/26.7	
Non-one child	135/57.9	148/73.3	
Missing	0	0	
Migrant status			.72
Migrant students	50/21.5	50/24.8	
Local students	182/78.1	150/74.3	
Missing	1/.4	2/.1	
Positive Youth Development	4.46/.66	4.25/.73	3.09**
***Familial factors***			
Family intactness			7.09**
Intactness	222/95.3	177/87.6	
Non-intactness	10/4.3	22/10.9	
Missing	1/.4	3/1.5	
Father’s Education Level			9.26*
Less than or middle school	76/32.6	80/39.6	
High school or college	79/33.9	63/31.2	
University	41/17.6	29/14.4	
More than university	28/12.0	9/4.5	
Missing data	9/3.9	21/10.4	
Mother’s Education Level			4.75
Less than or middle school	97/41.6	95/47.0	
High school or college	69/29.6	55/27.2	
University	41/17.6	22/10.9	
More than university	18/7.7	11/5.4	
Missing data	8/3.4	19/9.4	
Family Function	3.85/.77	3.57/.81	3.75***

*p < .05, **p < .01, ***p < .001

## Discussion

The present study investigated the prevalence of persistent depressive symptoms across 12 months among Chinese junior high school adolescents and simultaneously evaluated the influences of personal and familial factors on persistent depressive symptoms. In the present study, nearly half of the initially self-reported depressed students remained depressive during the course of a year. The prevalence of the persistent depression in this study is generally comparable with those reported by previous Western studies on adolescents [40–42]. The prevalence in the present study was relatively lower than that reported by Li et al. [12], possibly because the period of assessment in Li et al.’s study was shorter (9 months) compared to ours. Taken together, data collected from schools located in Shenzhen City generated results, which seem to coincide with those in the developed Western countries or in other economically equivalent areas (like Guangzhou city). More cross-cultural longitudinal studies are needed to substantiate the aforementioned intriguing findings associated with the level of economic development. The results of the present study may be primarily attributable to the fact that students have been in a more competitive academic environment [43]. It is widely recognized that persistent depression is associated with a range of mental illnesses like substance abuse, self-injury, and suicidal behavior [44] and has posed unique challenges to society. As such, Chinese society urgently needs researchers, educators, parents, and policymakers to work together to adopt effective intervention programs to address the issue.

Concerning demographic factors, having siblings was found to significantly predict persistent depressive symptoms over 1 year. Specifically, adolescents from one-child families were more likely to remit from depressive symptoms over time than adolescents with siblings. In view of the resource dilution model, in single child families, greater parental resources such as attention, time, and energy provided by parents of single children may lead to better parental guidance and individual care. Such social support may help depressed children make better psychological and behavioral adjustments, which provides greater opportunity to remit from depression [45]. Moreover, the single child group promoted by the one-child policy is embedded in a unique cultural, social, and economic background that has historical causes. According to Huang et al. [46], single children in China who generally live in areas with a more developed economy have parents with a higher level of education, and therefore a better occupational background such as being staff members of a state-owned enterprise, or in government departments and institutions. These children, who are likely to be living in a Chinese registered residence - known as “Hukou” have the opportunity to experience a richer and more diverse extracurricular life and thus a more pleasant childhood, than those from rural areas. These factors may serve to protect their mental health, thereby reducing the likelihood of persistent depression.

The results of this study also showed that adolescents with a lower level of positive youth development were more likely to develop persistent depression. Such results are supported by developmental psychopathology framework and previous studies in western cultures [47–49]. Thus, promoting positive youth development plays an essential role in protecting against depression. Under this condition, a number of interventional studies were conducted in North America and in Hong Kong, suggesting that positive youth development programs are associated with reduced problematic behaviors including depression, and additionally promotes positive development [23, 50, 51]. Moreover, several evidence-based interventions are found to be useful for prevention and treatment of persistent depression among adolescents, which include Treatment for Adolescents with Depression Study (TADS) and The Penn Resiliency Project (PRP) [52]. These interventions comprise of psychiatric and psychological components promoting mental health and addressing mental disorders among adolescents. However, such interventions are not widely adapted and implemented in the sociocultural context of China, which makes it unclear if these programs are also effective for addressing persistent depression or other disease-specific conditions in this unique population.

Regarding the familial level, students who lived in non-intact families are more likely to report persistent depressive symptoms. Results indicate that non-intact family and greater paternal-only educational attainment may respectively be risk and protective factors for depression. Parental divorce or death can be very stressful life events for children [53, 54], and make it extremely difficult for depressed children to recover [55]. In addition, initially depressed adolescents whose fathers had higher educational levels (bachelor’s degree and above) were more likely to remit from depression within 1 year. This is consistent with previous cross-sectional studies investigating the association between depression and parental educational level among Chinese children and adolescents [22, 56]. One explanation is that fathers with higher educational levels are more aware of the significance of a father’s influence and are more willing to put emotional and intellectual investment in parenting [22]. Conversely, fathers with low educational levels tend to adopt undesirable parenting strategies such as scolding and punishing [57]. Another possibility is that fathers with low educational levels are often busy with their livelihoods; many often have to travel far to work, or are away from home all year round. Thus, it may be difficult for them to have the time, energy, or resources to interact with their children [58]. These factors may increase the likelihood of mental health problems including persistent depressive symptoms in adolescents.

Further, poor family functioning strongly predicted a higher risk of persistent depressive symptoms, and adolescents from healthy functioning families were more likely to recover from depression. The results were similar to many cross-sectional and longitudinal studies, which have shown that healthy family functioning (e.g., strong family involvement, and open affective expression and communication) predicts fewer adolescent depressive problems [59, 60]. However, dysfunctional family environments, such as those offering less support, more conflict, and less cohesion, are correlated with more depressive symptoms. Researchers have shown that poor family environments can lead to strong negative emotions in adolescents (e.g., anger, hostility, self-blame, and helplessness) [61]. In addition, in negative family environments adolescents may directly or indirectly receive negative feedback from family members, prompting them to adopt a negative view and form negative self-perceptions [62]. Such negative emotions and self-perceptions may increase the risk of persistent depression among adolescents. Findings in our present study indicated that even though adolescents suffered from depressive symptoms, those who had a positive family environment were more likely to recover.

Several limitations of this study must be acknowledged. First, the CES-D scale was used to measure depression with a sum score of more than 16 points being considered a depressed level. Given that this self-reported questionnaire is commonly used in general populations, future studies on this similar topic that additionally include diagnostic instruments administered by clinical psychologists or psychiatrists are encouraged. Second, students with CES > =16 in this study were not referred to mental health services. In future studies, researchers could consider offering parents and students the option to be approached if the student screen positive using the CES-D to facilitate early diagnosis and treatment. Third, adolescent depression symptoms were only examined at two time points over a one-year interval. The categorization of remission versus persistent depression may only reflect the students’ status at one point in time and may be unstable. Future studies may explore the long-term developmental trajectories of depression in adolescence, by collecting more waves of data over a longer period, such as over the six-year period from Grade 7 to Grade 12. Fourth, the self-reporting approach taken by this study may be a limitation; adolescents may under-report their depressive symptoms because of social desirability. Future studies could include multiple reporting approaches, such as parent, teacher, or peer reports, to make the evidence more convincing. Fifth, the predictive factors of persistent depression in this study only include demographic information, a psychological factor (positive youth development) and family factors. According to the developmental psychopathology framework, family history of mood disorders (e.g., depression) and psychological vulnerability factors (e.g., childhood adversity and personality traits as neuroticism) were significantly correlated with persistence of depression [16]. Future research may contain multiple factors to provide a more comprehensive picture of persistent depression in Chinese adolescents. Finally, as the study was conducted in junior high schools in Shenzhen, a modern city of China, and the data in the rural area were unavailable, the results may not be generalizable to adolescents across China.

## Conclusion

This study has several theoretical and practical implications. Concerning theoretical implications, this study adds to the knowledge on persistence/remission of depression through a longitudinal study of Chinese adolescents. Besides, the prevalence and predictors of persistence and remission of depression are important components of a developmental and psychopathological perspective of adolescent depression in China [8, 63]. Regarding practical aspects, we found that, firstly, the prevalence of persistent depressive symptoms among adolescents in China is relatively high. This suggests that effective strategies to help youth prevent and reduce depression must be developed and implemented in order to promote mental health. Secondly, adolescents from families with siblings and with low levels of positive youth development need particular help to prevent and reduce the possibility of persistent depressive symptoms. The promotion of positive youth development in adolescents could be a promising direction in preventing/reducing persistent depression among adolescents in China. Thirdly, family-based approaches that pay attention to enhancing parenting capabilities and family relationships by raising understanding, communication, and warmth and reducing conflicts between parents and children, may provide a way for preventing or reducing persistent depression among the adolescents. Moreover, for the families where fathers are with low level education, the communities and schools may carry out workshops or activities about how to improve fathers’ involvement with their children’s development and academic achievement [64]. For example, these fathers may be encouraged to talk to his children about school life, communicate with teachers, participate in activities organized by the school, and have a reasonable expectation for his children [65].

## Data Availability

The dataset used in the analysis of this study is available upon reasonable request and with permission of Shenzhen University.

## Abbreviations

CI: Confidence interval
PYD: Positive youth development
CES-D: Epidemiologic Studies Depression Scale
CPYDS: The Chinese Positive Youth Development Scale
CFAI: The Chinese Family Assessment Instrument
TADS: Treatment for Adolescents with Depression Study
PRP: The Penn Resiliency Project
